# Clinical genetic services in the Emilia-Romagna region, Italy: current activity and open issues: a mixed-method study

**DOI:** 10.1007/s12687-024-00750-7

**Published:** 2025-01-11

**Authors:** Lea Godino, Enrico Ambrosini, Valeria Barili, Claudio Graziano, Livia Garavelli, Olga Calabrese, Marcella Neri, Luca Sangiorgi, Benedetta Bertonazzi, Giovanni Innella, Daniela Turchetti, Antonio Percesepe

**Affiliations:** 1 Medical Genetics Unit, IRCCS Azienda Ospedaliero-Universitaria di Bologna, Bologna, Italy; 2 Medical Genetics Unit, University Hospital of Parma, Parma, Italy; 3 Medical Genetics, Department of Medicine and Surgery, University of Parma, Parma, Italy; 4 Medical Genetics Unit, AUSL Romagna, Cesena, Italy; 5 Medical Genetics Unit, Azienda USL-IRCCS of Reggio Emilia, Reggio Emilia, Italy; 6 Medical Genetic Unit, University Hospital of Modena, Modena, Italy; 7 Medical Genetics Unit, Department of Mother and Child, Sant’Anna University Hospital of Ferrara, Ferrara, Italy; 8 Department of Rare Skeletal Disorders, IRCCS Istituto Ortopedico Rizzoli, Bologna, Italy; 9 IRCCS Azienda Ospedaliero-Universitaria di Bologna, Bologna, Italy; 10 Department of Medical and Surgical Sciences (DIMEC), University of Bologna, Bologna, Italy

**Keywords:** Genetic counselling, Workforce, Staffing, Mixed-method design

## Abstract

In 2002, in the Emilia-Romagna region of Italy, a comprehensive strategic plan was developed with the aim of improving the integration and efficiency of the genetic services. Two decades later, this report aims to explore the current functioning of the regional network, with special focus on clinical genetics in the evolving scenarios. To this aim, we analyzed the activity data of the medical genetics services in the region, to identify and possibly improve currently open issues. This is a mixed-method study, analyzing quantitatively and qualitatively the activities of seven medical genetics services in Emilia-Romagna region. Quantitative analysis considered the number of consultations and the composition of the staff in the year 2021. Qualitative analysis examined a focus group of directors of the services through reflexive thematic analysis. A total of 14,925 counseling sessions have been delivered by the medical genetics services, staffed with 22.4 full-time equivalent clinical geneticists. A physician performed an average of 14.5 consultations per week and approximately 1166 h of patient care per year. The clinical geneticists/inhabitants ratio was 0.54 per 100,000 inhabitants, and it is estimated that one every 278 inhabitants, on average, underwent a genetic counseling session in 2021. Qualitative analysis highlighted issues concerning patients’ access to service, general organization and staff composition. In order to meet the growing demand for genetic counseling services, expansion of the workforce and adjustment of current practice models are required to increase the access to genetic services and the application of test results to clinical management.

## Introduction

The last decades have witnessed a widespread diffusion of genetic testing, with an estimated annual increase of referrals to the clinical genetic services between 23% and 25% (Blout Zawatsky et al. 2022; Dragojlovic et al. 2020). The same growth rate is not reflected in an equivalent increase in the number of professionals in charge of providing those genetic counseling sessions. Several authors have pointed out the overwhelming workload and the scarcity of health workers with the specific background (Blout Zawatsky et al. 2022; Maiese et al. 2019). A recent scoping review has described the composition of the clinical genetics’ workforce in high-income countries and has highlighted the requirements for delivering a comprehensive patient care in Clinical Genetics, identifying three primary professions as fundamental components of the multi-professional team: clinical geneticists (specialized physicians), clinical scientists (laboratory biologists or biotechnologists), and Genetic Counselors or Genetic Nurses (Dragojlovic et al. 2020).

In 2002, a comprehensive strategic plan was developed in the Emilia-Romagna Region, a highly industrialized, high-income, densely inhabited area of Northern Italy (Delibera della Giunta della Regione Emilia-Romagna No. 1267, 22.7.2002. http://www390.regione.emilia-romagna.it/webattn/aw01w51s.). Before that, a formal organization of genetic services was lacking in Emilia-Romagna and in many other regions of Italy. The aim was to improve the integration and efficiency of genetic services, including clinical genetics and laboratories, allocating specific tests to single molecular laboratories in order to promote mutual relationships and enable their diversification and specialization. A hub and spoke model was adopted, considering four molecular laboratories affiliated with medical faculties as the hub for molecular genetic activities and two main laboratories serving the northern and southern part of the Region, respectively, as the hub for cytogenetic activity, whilst a single center constitutes the hub of clinical genetics. One of the issues raised at that time was the lack of genetic counselors in the genetic teams, reflecting their absence in the Italian health care system (Calzolari et al. 2005).

In the subsequent years, some national surveys of medical genetics services have been undertaken by other groups, sponsored by the Italian Society of Human Genetics (SIGU), the last one of which refers to the year 2011 (Giardino et al. 2016). This survey included also private facilities and showed that total medical genetics services were actually abundant, disproportionate to the population and not equally distributed across the country, with a decreasing gradient towards the south.

The Emilia-Romagna Region, located in northern Italy, is one of the largest regions in the country, with 4.460.030 inhabitants, of which a significant percentage (12.8%) are non-Italian native (Regione Emilia-Romagna 2023). From the genetic point of view, there are founder effects described in literature (Zuntini et al. 2017) and specific areas with a high prevalence of beta thalassemia carriers, which translates in public health measures (Gallerani et al. 1990).

Two decades after the regional plan was undertaken, there is still need to understand the current functioning of the regional network, with special focus on clinical genetics in the evolving scenarios. To this aim, we analyzed staffing and activity data of the clinical genetics services in our Region, to identify critical issues and to propose possible improvements.

## Methods

### Study design

This study employed an exploratory mixed-method approach, incorporating both quantitative and qualitative analyses. The quantitative and qualitative components were not conducted in a strictly chronological sequence; rather, they complemented each other to provide a comprehensive overview of the Genetics services in the Emilia-Romagna Region. There are a few other public entities providing genetic counseling sessions, mainly in the oncology field or inside family planning clinic, but we chose to consider only autonomous clinical genetics services primarily led and composed by clinical geneticists.

### Quantitative phase

#### Data collection

For each clinical genetics service, the following data were collected:

- number of genetic counseling sessions performed over one year (namely, 2021). We decided to take the year 2021 as the reference year because the activities of that year had been already collected and subjected to assessment by the Italian Ministry of the University in the framework of the accreditation for Postgraduate Courses in Medical Genetics;
- number of medical geneticists, biologists, nurses, and other professionals who were actively working in the same year. Many studies applied to workforces utilize the term “Full Time Equivalent” (FTE) to indicate an individual who dedicates the totality of his/her working time to that specific mansion. Applied to our situation, we considered 1 FTE for physicians hired by the hospital, 0,6 FTE for those working for the university (since national laws define that 1/3 of their time must be dedicated to research and teaching) and other fractions for part-time workers. Other healthcare professionals were not included in the workforce assessment since they are not formally recognized by the Italian healthcare system so their work in this context is under the responsibility of the referring physician. For example, medical residents are surely contributing to clinical practice, but they are considered as university workers (not hired from the Hospital) and are not independent in their clinical or laboratory activities.

#### Genetic counseling protocol and assessment of the workload

Genetic counseling was mainly delivered through in-person sessions, although there were occasional instances of teleconsultations.

Generally, a clinical geneticist leads genetic counseling sessions, sometimes assisted by clinical geneticists in training and, rarely, by nurses. The genetic counseling sessions followed the standard care protocol, with the first consultations including a comprehensive gathering of family history, pedigree drawing, analysis of clinical records of index case and affected family members, clinical examination, discussion of diagnostic hypotheses and of significance of genetic testing for the proband and the family, if appropriate, and presentation of options for risk reproductive management. Subsequent genetic counseling sessions post-testing involved the disclosure of test results, clinical interpretation, and the formulation of management plans.

The Italian Society of Human Genetics (SIGU) prepared a specific document on the workload of the clinical geneticists which delves into the specific tasks and responsibilities assigned to the clinical geneticists, highlighting the intricate nature of their work, and directing the distinction between simple and complex genetic counseling sessions depending on the clinical field. According to this document, the mean time-length required for complex sessions is estimated as high as 2–3 h (including case evaluation and literature/database searches beyond the time of the visit), while for simple sessions the time requested is 40–50 min on average (SIGU 2018).

Since the data collected did not allow a clear distinction between the two types, we decided to consider an average time of 105 min per session, which is consistent with the time-length defined in other papers, although others indicate even longer time slots especially for pediatric evaluations (McPherson et al. 2008; Sukenik-Halevy et al. 2016; Surh et al. 1995).

### Qualitative phase

#### Interviews collection

A focus group with Directors of the clinical genetics services was arranged at the end of a meeting of the Emilia-Romagna section of the Italian Society of Human Genetics, held in January 2024 in Bologna. Of the six Directors invited, five participated in the focus group, either in-person or online. The 3-hours discussion, facilitated by one of the other authors (not a director), aimed to explore the experiences within different services, including available resources, the time required for genetic counseling, the professionals involved and interactions with other healthcare professionals. The discussion was intentionally held in an open format to encourage interaction among participants. The focus group was recorded and transcribed using the Teams platform.

#### Data analysis

The qualitative phase utilized reflexive thematic analysis (Braun and Clarke 2006, 2019). This approach, used to uncover both implicit and explicit ideas within the data, involves extracting themes. We opted for an inductive approach to explore the experiences of service directors, emphasizing the perspectives of professionals without being confined to a pre-established analytical framework.

We followed the six phases described by Braun and Clarke (2019). In the initial phase, familiarity with the data was established by taking notes during and after the interviews, capturing impressions and potential interpretations. After transcription, a thorough reading of all interviews was carried out by two different coauthors. The second phase involved detailed and systematic coding, utilizing constant comparative methods. Open thematic coding was applied to extract meaning from the data and organize it into codes. The third phase focused on constructing themes across the data based on research questions and researchers’ interpretations. Tentative themes and sub-themes were outlined, considering patterns and statements in the text. Some statements were initially categorized under multiple themes due to perceived overlaps. In the fourth phase, themes were discussed and revised to eliminate overlaps and clarify relationships. The fifth phase involved defining and naming themes more precisely to convey the essence of the empirical data. The final phase consisted of checking the consistency and appropriateness of the selected themes. In this phase, a third author (DT) was involved. Thematic analysis was conducted on Italian-transcribed data, with the subsequent translation of themes, codes, and quotes into English.

#### Rigor

Rigor was ensured by the use of robust qualitative research methods (Neergaard et al. 2009; Sofaer 1999), adhering to established standards for qualitative research (Tong et al. 2007), and utilizing techniques to enhance rigor (Barbour 2001; Braun and Clarke 2019) throughout the transcription, coding, analysis, and report creation processes. Specifically, we ensured detailed transcription through audio recording verification, and independent coding by two authors assured equitable consideration of all data. Additionally, a re-analysis of the original data set post-coding was conducted to collate all coded items and verify all themes (Braun and Clarke 2019).

## Results

### Clinical genetics services and staffing

**Fig. 1:**
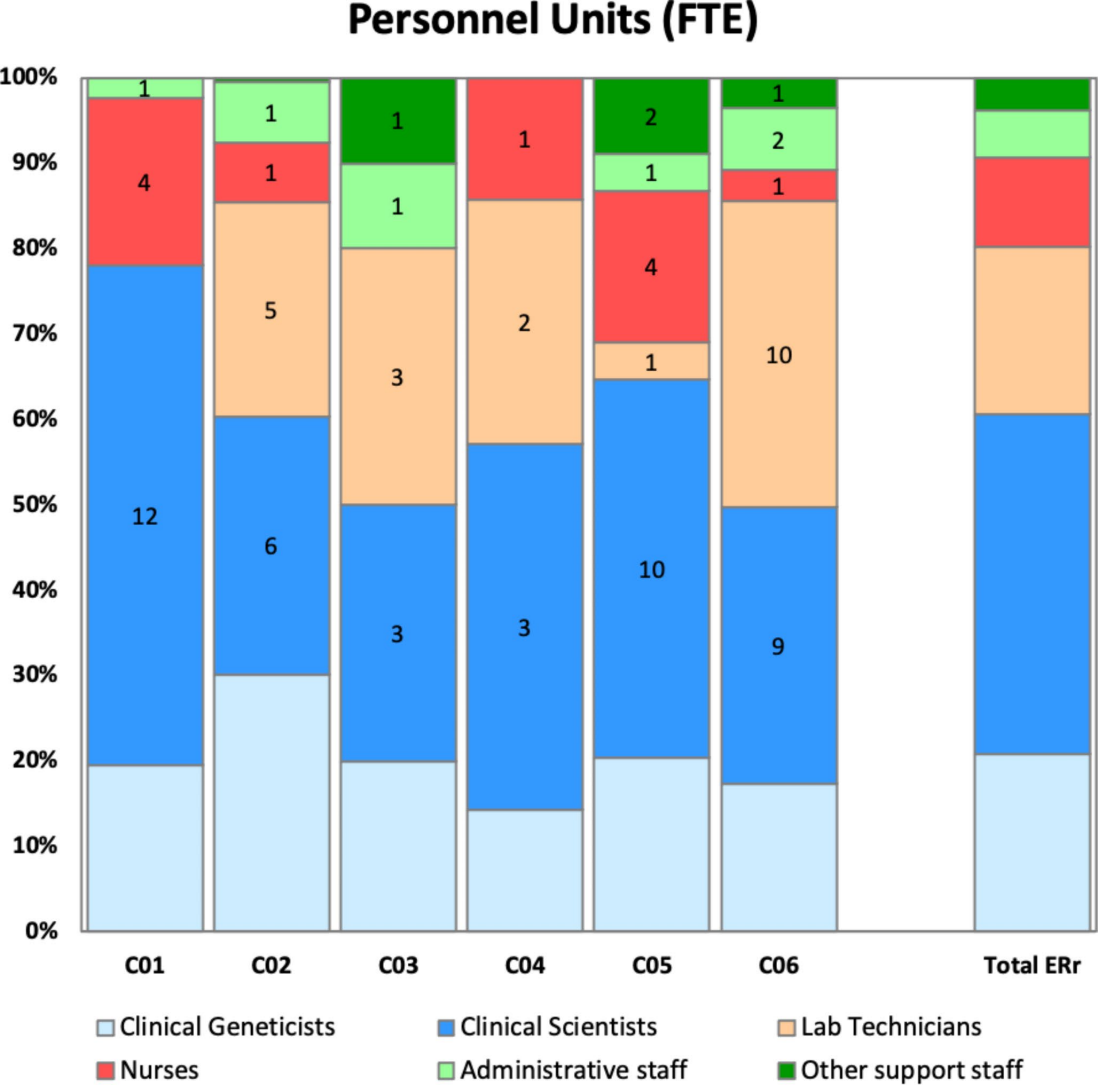
Composition of the staff of the six clinical genetics services (FTE = Full Time Equivalent). The absolute numbers are inside the colored bars, while percentage is on the y-axis. The codes on the x-axis indicate the different clinical genetics services. As explained in the text, medical residents and staff from highly specialized centers were excluded

In the period 1.1.2021–31.1.2021, the Clinical Genetics services were operating in six areas of the region, employing a total of 25 certified clinical geneticists (22.4 Full Time Equivalents, FTE, corresponding to an average of 0.54 FTE every 100.000 inhabitants, see also Table 1), together with 43 FTE Clinical Scientists (Biologists/Biotechnologists), 35 FTE lab technicians, 11.4 FTE Nurses, 5.9 FTE Administrative staff and 4.1 FTE other support staff (Fig. 1). The seventh service, initially involved, was not included in the calculations since it is a specialized center for skeletal diseases and is not directly associated to a specific geographic area. As explained in the Methods section, medical residents were excluded from the official count: nevertheless, in order to have a more comprehensive overview, we report that, in the same time frame, a total of 17 residents (not shown in Table 1; Fig. 1) were participating in genetic counseling throughout the training network.

**Table 1:** Number of professionals involved in genetic counseling related to the population (MD = medical doctors specialized in Clinical Genetics, residents are not included)

Services	Inhabitants^1^	Number of MD ^2^	MD per 100k inhabitants	Other professionals (non-MD) directly involved in genetic counseling sessions
**C01**	1,117,062	4.0	0.4	1 clinical scientist
**C02**	527,140	6.0	1.1	6 clinical scientists
**C03**	449,628	2.0	0.4	3 clinical scientists
**C04**	703,696	1.0	0.1	1 nurse
**C05**	342,061	4.6	1.3	2 nurses
**C06**	1,015,608	4.8	0.5	1 nurse
**TOTAL**	**4**,**155**,**195.0**	**22.4**	**0.54**	**10 clinical scientists + 4 nurses**

^1^ In the year 2021. ^2^ Full Time Equivalent (FTE), see Materials and Methods section

Three of the centers (C03, C05, C06) belong to academic hospitals and contribute to teaching activities of Medical, Nursing, Biology and Biotechnology Schools. Moreover, two of them (C05, C06) coordinate Postgraduate Residency Programs of Medical Genetics, whereas all the hospitals participate in the Residency network, hosting variable numbers of clinical geneticists in training.

While all the clinical geneticists are involved in genetic counseling, involvement of other professionals, namely clinical scientists and nurses, is still limited. Selected biologists are performing short pre-test genetic counseling sessions for screening tests (e.g. cystic fibrosis or karyotype in IVF programs) in C01, C02 and C03. Nurses mostly collaborate for sample collection and patient registration, but they are actively employed in selected genetic counseling sessions (e.g. pre-testing genetic counseling in karyotypic analysis) in some centers (C04, C05, C06).

### Workload

In 2021, a total of 14,925 genetic counseling sessions have been delivered by the clinical genetics services of Emilia-Romagna, including both the consultations for new patients and the follow-up visits (see Limitations). It can be approximated that most of them are new patients, since follow-up visits are only prescribed for selected patients who previously underwent genetic testing.

In terms of time, the average annual clinical workload per physician is 1166 h, with an average of 14.5 consultations per week. Approximately one out of 278 inhabitants in Emilia Romagna received a genetic consultation in 2021 (Table 2).

**Table 2:** Number of genetic counseling sessions compared to the medical staff* and the inhabitants of the six areas covered by the clinical genetics services in Emilia-Romagna (*Other healthcare professionals were not included in this calculation because their work in this context is under MD responsibility)

Service	Inhabitants	Counseling sessions	Time spent per MD (h)	Sessions per year per MD	Average Sessions per week per MD	Sessions / inhabitants ratio
**C01**	1,117,062	3716	1625.8	929	20.2	1:301
**C02**	527,140	2794	814.9	466	10.1	1:189
**C03**	449,628	1604	1403.5	802	17.4	1:280
**C04**	703,696	710	1242.5	710	15.4	1:991
**C05**	342,061	3184	1211.3	692	15.0	1:107
**C06**	1,015,608	2917	1063.5	583	13.2	1:348
**TOTAL**	**4**,**155**,**195**	**14**,**925**	**1166.0**	**646**	**14.5**	**1:278**

### Qualitative phase

**Fig. 2:**
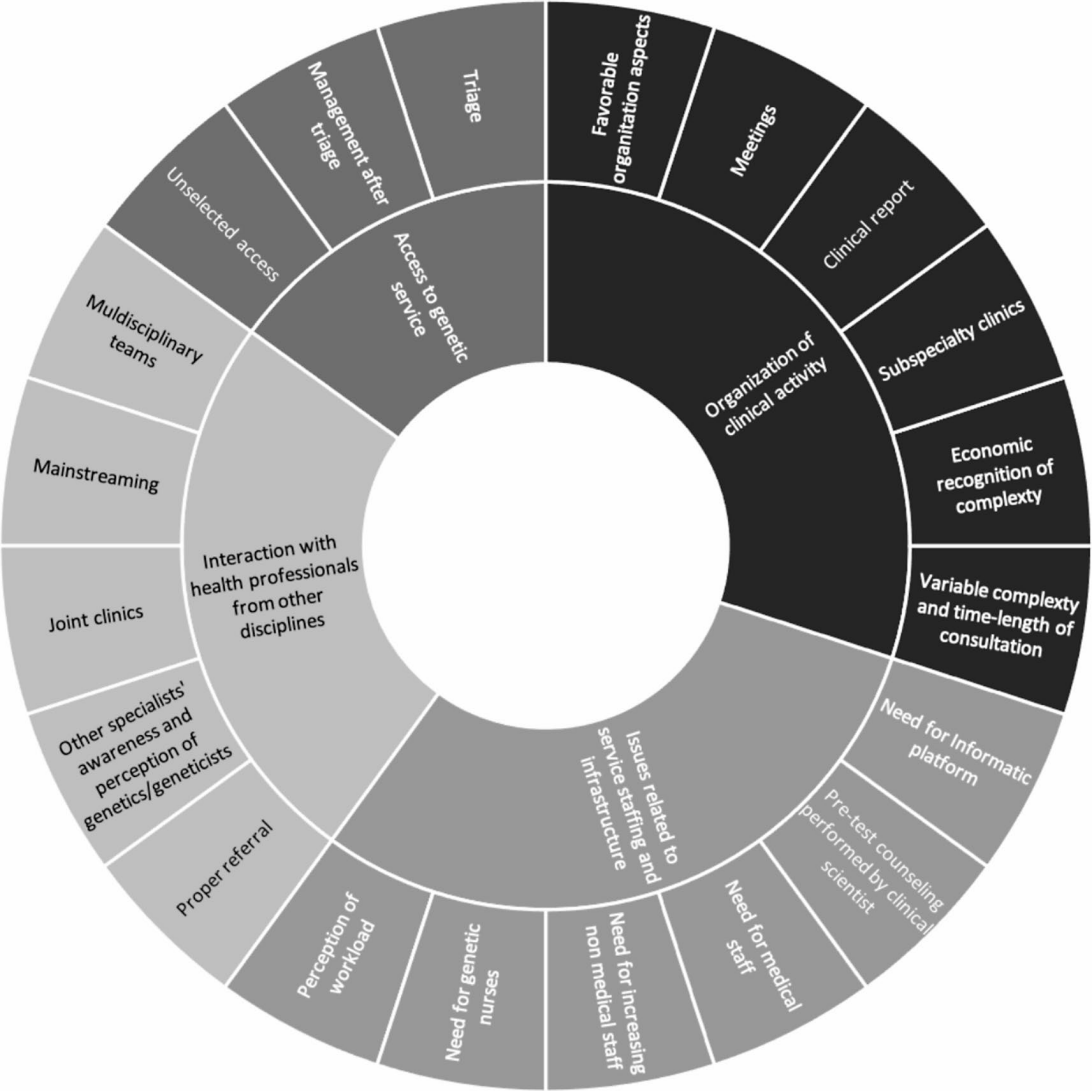
Results of the qualitative analysis

The directors of five out of six Clinical Genetics services agreed to participate in the Focus Group: four (C01, C03, C04, C06) in-person, the other one (C03) online through the Teams platform. As reported in Fig. 2, the discussion revolved around the following major themes (Fig. 2):

1. Access to genetic services.
2. Organization of clinical activities.
3. Interaction with health professionals from other disciplines.
4. Issues related to Service staffing and infrastructure.

#### Access to genetic services

In the focus group, access to genetic services was discussed, with special regard to the opportunity of screening requests and pre-select clients for whom genetic evaluation is appropriate and clinically useful.

Indeed, a meaningful proportion of referrals is regarded as not appropriate, which impacts on the efficiency of services; this seems particularly common among self-referrals:

…at least half of self-referrals for cancer genetic counseling are inappropriate….

…there are people requesting counseling because they have been diagnosed with celiac disease and Hashimoto thyroiditis […] I wonder whether it has been explained by gastroenterologists and endocrinologists [that those are not mendelian diseases]….

Among participants, two reported to systematically perform preliminary evaluation of the requests to select those that are appropriate, while in the other services the selection is not standardized or is limited to specific request types (urgent prenatal consultations).

Triage may be performed through a structured way, with self-referring patients required to fill in specific forms and provide clinical documentation:

… each geneticist oversees a subspecialty clinic (pediatrics, cancer, etc.) and assesses respective request forms and clinical documentation….

Alternatively, requests are discussed in staff meetings, but assessment is made by an expert of the subtype as well:

We assess the requests every Friday morning. Each of us has expertise in one specific field….

Even in the absence of a triage procedure, the clinical geneticist is often requested to confirm the appropriateness of the referrals:

When the secretary is unsure, she asks the nurses, who eventually ask me… it does not work properly; nurses should be more autonomous in triaging cases….

In C06, where triage is applied, patients who do not meet requirements for accessing consultations receive remote genetic counseling by a clinical geneticist:

… if a request is not appropriate, we call the person by phone […] every month, one of the clinicians – yes, unfortunately a physician – is on turn for telephone counseling. We then report in the hospital platform the reason why we did not deliver the consultation requested….

‘…we perform a videoconsultation…’.

In several services, a proportion of appointments for genetic consultations is made through a centralized booking system (“CUP”) of the area, which citizens use to arrange medical examinations without any prior selection.

#### Organization of clinical activities

Another theme of discussion was the organization of clinical activities: a relevant impact of variable complexity and time-length of different consultation types were generally acknowledged:

Sometimes a consultation takes one-hour, other times two hours, then you have to study the case further […] many are complex consultations, especially the pediatrics ones!

…in consultations on reproductive matters, there are two consultants for one visit, and you need to collect both pedigrees.

…those planned by CUP [centralized booking system] are more likely to be inappropriate requests, and need no more than 15 min….

The complexity of consultations is not paralleled by adequate financial recognition in the public healthcare system:

…the fact that there are only two fares (first consultation and follow-up), both very low, means that complexity and time-consumptionn are not properly valued!

Timing of clinical report provision was also a matter of discussion:

‘We check the clinical records before the consultation and prepare a draft […] however after examining the patient a differential diagnosis may be needed, implying much more work to study the case; we may give the family a provisional report, or waiting to have completed the evaluation to give the final report’.

Among the strengths of the organization, the co-existence with a genetic laboratory allowing multi-professional case discussion and the ability to offer various types of consultations, with dedicated clinics, were reported:

…the implementation of dedicated clinics is progressively increasing and reflects the multiplication of medical branches interacting with the Genetics: this is the evolution….

#### Interaction with health professionals from other disciplines

A relevant part of the focus group was pointed on the interaction with health professionals of other disciplines; issues raised included proper referral, awareness/perception of genetics/geneticists and establishment of multidisciplinary paths.

Beyond self-referrals, even referrals from other specialists may pose issues:

…some cardiologists refer hundreds of elderly patients with cardiomyopathy [occupying all the available slots], then complain because the first appointment available for a 30-year-old patient is after one year….

Nevertheless, the issue of what types of consultations should be deemed as appropriate was raised in the discussion, eliciting different views.

Someone was in favor of broader access criteria:

A person who is worried because of a family history of colon cancer has the right to receive counseling.

…even for a common condition such as autism, clinical genetic assessment may reveal clues for a syndrome diagnosis, so it is preferable to see as many cases as possible….

Others would prefer stricter selection:

‘For very common situations, other health professionals should be in charge of giving proper information […] our role should be to promote knowledge and raise awareness, not to deliver counseling to each individual’.

In any case, instances of scarce awareness about the role of geneticists and the complexity of genetic tests among other specialists were unanimously reported:

‘Cardiologists and Neurologists may be surprised by the time-lapse between referral and test results; after seeing patients and refer them for a genetic consultation, they expect that results of genetic investigations are immediately available for them to conclude their assessment’.

‘There is not yet enough knowledge of our discipline’.

However, it was suggested that working together with geneticists in multidisciplinary paths may lead other physicians to recognize their role and the added value of integration:

‘At the beginning many clinicians do not understand our role: genetic counseling seems an unnecessary step, which complicates and delays the route to genetic testing; later on, when they have to face challenges in clinical interpretation of test results, communication to other family members and so on, they recognize the value of our discipline: we need to interact more to change their mind, their prejudices’.

The value of multidisciplinary paths was recognized by all participants; someone highlighted the value of joint clinics and multidisciplinary assessment of patients:

…often children need to be seen by clinical geneticists and neuropsychiatrists together, otherwise some diagnoses may be missed….

We run a joint clinic with Gastroenterologists for individuals at familial risk of digestive cancers: even if there are no criteria for genetic testing, they can enter a surveillance program….

‘We meet on a regular basis with every multidisciplinary team we have joined’.

For some disorders, mainstreaming approaches were deemed highly efficient:

…we must collaborate with other specialists […] there is no reason for us to see every single patient with pheochromocytoma, who undergoes genetic testing in any case.

‘We should establish multidisciplinary paths and work mostly in integrated care pathway sharing criteria for genetic testing, which can be arranged by other specialists: we should see only patients with variants detected or with suspicious features and discuss complex cases in multidisciplinary meetings’.

#### Issues related to service staffing and infrastructure

Compared to the number of consultations registered, perceived workload was much higher, since several activities, such as triage and telephone genetic counseling, that are carried out by clinicians fail to emerge in quantitative assessment; thus, all felt that the number of clinicians in the services is undersized for the work required:

Triage is energy- and time-consuming. It constitutes a heavy load for clinical geneticists.

Even one more doctor would be of help….

There is too little time for in-depth study of cases and for research….

Other staff is of limited help for genetic counseling activities:

For specific tests, pre-test counseling is targeted and is delivered by a clinical scientist, who provides clients with an information sheet and some explanations, but no more….

…our nurses oversee sample collection and mailing […] we would need nurses trained in genetics, who may contribute to counseling activities….

‘…we would need a specialized nurse to manage the cases…’.

Lack of administrative and technological support is felt to increase the non-clinical workload of physicians:

A lot of work is administrative in nature, and should be made by administrative staff, who in reality is largely inadequate in terms of numbers.

‘If we had more processive informatic platforms supporting clinical work, test requests, and so […] we would save time and energies…’.

## Discussion

More than twenty years have passed since the start of the implementation of the strategic plan for the organization of medical genetics services in Emilia-Romagna. In this period of time, the integration of genetic services into the medical mainstream has not been uniform throughout Italy, due to the delegation of health care organization to regional authorities, as shown by three national surveys of medical genetics services in Italy, performed from 2004 to 2011 (Dallapiccola et al. 2006; Giardino et al. 2016). In particular, from 2007 to 2011 there was an increase of more than 40 clinical genetics services, considering also private facilities, while there was a reduction of the cytogenetics laboratories, possibly due to an initial change of the prenatal aneuploidy screening procedures. More recent data can also be obtained from the accreditation of Medical Residency Programs: in order to obtain this accreditation, a public medical genetics service has to satisfy strict parameters, mainly related to quantitative measures of counseling sessions, genetic tests performed and working staff (visible on the official Ministery of Education website, https://www.mur.gov.it). Until 2016, only 8 Residency Programs specific for Medical Genetics existed in Italy, while in 2021 the number increased to 21, distributed in almost every region,.Some region adoptied a more centralized approach, having only one hub laboratory performing genetic tests for all the provinces, whereas others developed a multicentric network of laboratories.

Another essential factor to consider is the important technical advancements of the last 20 years, which indirectly led to an increase in the requests of genetic tests. In Emilia-Romagna, cytogenetics and single gene approaches (e.g. Sanger sequencing, PCR) were prevalent up to 2014, like in other regions (Giardino et al. 2016). Starting from 2015, all the genetic services involved in this study gained access to Next Generation Sequencing, performing tests solely in a public setting, without the involvement of private partners.

Concerning the personnel, the 2011 Italian survey reported a total of 3246 persons employed in the national genetic services, of which 38% biologists, 21% technicians, 16% medical doctors, 9% administrative, 7% healthcare personnel, 6% biotechnologists, 1% bioinformaticians, 1% graduated in different disciplines, and 2% other staff (Giardino 2016). These numbers are similar to the ones identified in our study, confirming the higher number of biologists as compared to specialized physicians. Interestingly, in the 2011 survey only 12% of genetic tests were followed and/or preceded by genetic counselling, thus not fulfilling the institutional indications and explaining, according to the authors, the low rate of appropriateness detected (Giardino et al. 2016).

Our study shows that multiple issues still need to be addressed for genetic counseling and testing to be widely available and maximize their clinical impact, including the supply of professionals with genetics expertise and knowledge for non-geneticist providers. Pediatric assessment may need a joint effort of geneticists and other specialists like neuropsychiatrists, whereas adult-onset conditions with a higher population frequency sometimes determine an unnecessary burden on understaffed genetic services, with significant delays on access to testing. Triage procedures could be effective in this respect, but require significant effort, as emerged from the qualitative part of this study. Patients not meeting the criteria for in-person genetic counseling may be managed through telephone counseling, which is also a time-consuming activity in the absence of professionals other than physicians; in addition, it is generally not formally recognized in financial terms by the public healthcare system.

On the other hand, uncontrolled genetic testing, without filters or at least feedback from genetic specialists, could lead to inappropriate exams, resources consumption and a higher impact of inconclusive or incidental findings (Elliot et al. 2021). Moreover, several studies have demonstrated the cost-effective and timesaving benefits of mainstreaming approaches both in the oncological field (Scheinberg et al. 2021) and in adult chronic diseases (Elliot et al. 2021). These models have been pursued by some of the genetic services of our Region: pre-test genetic counseling with consent and sample collection have been performed by a physician (oncologist, nephrologist) not specialized in Medical Genetics, but with a proper education in the specific field and a constant follow-up with a geneticist; genetic counseling has been reserved to more complex cases, and to interpretation and communication of positive or uncertain results, as well as to assessment of other family members. Some centers have developed multidisciplinary teams for Oncogenetics, Neurogenetics, Cardiogenetics or other joint services, with only partial and often indirect involvement of the medical genetics service, but the modality of access and organization is heterogenous throughout the region, so we chose to take into account, for all the statistics, only the activity performed by Medical Genetics specialists recruited by a medical genetics service.

This is, to our knowledge, the first population-based, regional study in Italy to assess the number of genetic counseling sessions and the composition of the staff of the services operating in this relatively new discipline, with the aim of exploring the ability to meet the rising need for genetic counseling services, driven by the advancements in genetic testing technology and the increased awareness among both healthcare professionals and patients (Cohen et al. 2012).

Indeed, a recent scoping review consistently highlighted that the present capability of the clinical genetics workforce falls short of satisfying the growing demand for genetic services (Dragojlovic e al. 2020).

The literature did not reach a consensus regarding the optimal ratios required to deliver adequate genetics services. However, the provider-population ratio was estimated at an average of 0.7 full-time equivalent per 100,000 inhabitants for genetic counselors and at 0.3 full-time equivalent per 100,000 inhabitants for clinical geneticists (Dragojlovic e al. 2020). Considering these data, it can be concluded that there is an expected presence of 2.33 genetic counselors for every clinical geneticist. Emilia-Romagna has an average of 0.54 FTE clinical geneticists per 100,000 inhabitants but no recognized genetic counselor or specialized genetic nurse. Although activities like medical examination, handling of complex cases, and making diagnoses are the exclusive responsibility of the clinical geneticists, most tasks in genetic healthcare can be performed by other professionals (Dragojlovic e al. 2020). Nevertheless, the regulatory framework for genetic counselors and genetic nurses is highly variable across countries; in a European survey, despite their absence in several countries, they were considered as the most important professionals after the physicians (Catapano et al. 2022). Genetic counselors and nurses are not yet recognized professionals in the Italian healthcare system, although recently the first Master Degree program in “Genetic Counsellors” has started at the University of Siena (https://genetic-counsellors.unisi.it/en).

The inclusion of these professionals is crucial, given the expected continuous increase in demand for genetic services. As envisaged in our study and in the literature, the main consequence of failing to adapt to this phenomenon would be the reduction in the ability of genetic services to complement some specific needs of the patients and their families. Besides, even from a “work/life balance” point of view, the lack of those professionals already increases the workload of the clinical geneticists, who often choose to work beyond regular hours, including evenings and weekends at home, to attend to more patients and engage in additional academic activities (Bernhardt et al. 2012; Calzolari et al. 2005).

The limitations of the study mainly reside in the lack of details on the type of genetic consultations: we only had access to the total number of consultations and attributed them an average time-length. However, a higher proportion of complex cases could involve an underestimation of the workload and their distribution across centers could account for some of the differences registered, as discussed in the focus group. The data regarding counseling activities performed by other clinical services (e.g. Oncology) are also missing, so the number of inhabitants involved in genetic counseling and testing is probably higher than what we stated.

The population data need to be interpreted with caution too, since the hospitals are relatively close to each other (average of 45 km distance, except for C01 that covers a vast area) and the patient can show up in other provinces or even other regions. Even in the absence of specific mobility data, it appears likely that the number of inhabitants going outside Emilia-Romagna for genetic counseling sessions is similar to the number of patients coming from adjacent regions, thus not impacting on the final results. Many previous studies had the limitation of being centered on single health centers, often with a high specialization for a group of diseases; in this study, we analyzed the data coming from all the different genetic counseling services of the Emilia-Romagna region.

## Conclusion

In order to meet the increasing demand for genetic counseling services, adjustment of current practice models seems to be required. This could include improved triage processes, greater involvement, and expanded responsibilities for genetic counselor/nursing professionals. Additionally, expanding the workforce and redistributing responsibilities may help alleviate the current workload faced by genetic professionals. Standardizing activities and implementing specialized nursing education programs in genetics, along with promoting formal recognition of these professionals in Italy, are also crucial steps in ensuring broader access to high-quality genetic services.

## Data Availability

All relevant data are available from the corresponding author upon reasonable request.
